# Surgical treatment of giant gist with acute gastrointestinal bleeding: Case report

**DOI:** 10.1016/j.ijscr.2018.11.021

**Published:** 2018-11-16

**Authors:** Catarina Melo, Carolina Canhoto, Fernando Manata, António Bernardes

**Affiliations:** General Surgery Department, Coimbra Hospital and University Center, Praceta Prof. Mota Pinto, 3000-075 Coimbra, Portugal

**Keywords:** Gastrointestinal stromal tumor, Emergency surgery, Gastrointestinal bleeding, Jejunal tumor

## Abstract

**Introduction:**

Gastrointestinal stromal tumors represent about 1% of the tumors of digestive tract. Their presentation consists often of indolent symptoms. However, they can present as surgical emergencies in rare cases. This work presents a case of a giant jejunal GIST that required emergent surgical treatment.

**Case presentation:**

A 60-year-old male presented with abdominal pain and acute low gastrointestinal bleeding. During observation a palpable mass was detected in the periumbilical region, left hypochondrium and left flank. A non-total colonoscopy was inconclusive. An Angio-CT showed a large exophytic tumor arising from the 4^th^ duodenal segment and first jejunal segment with approximately 20 × 14 x 13 cm, with apparent intratumoral bleeding. Due to haemodynamic instability, the patient was submitted to emergency laparotomy, in which a large tumor was found arising from the first jejunal segment. A segmental enterectomy was performed and the post-operative period ran without complications. The histopathologic exam showed a gastro-intestinal stromal tumor of epithelioid cell nature, with low mitotic count (<5 per 50HPF), categorized as T4N0 stage IIIA. The imunohistochemical analysis revealed positivity for CD117 (c-kit) and DOG-1. The patient started Imatinib therapy, and is free of disease recurrence for 3 years.

**Conclusions:**

This case pretends to review an unusual presentation of a giant jejunal intestinal GISTs as well as its management in an emergent context.

## Highlights

• Small bowel GIST is an unusual tumour of the gastrointestinal tract and can have various clinical presentations.
• Its presentation as acute gastrointestinal haemorrhage is unusual, and high index of suspicion is required for diagnosis.
• We describe a case of a giant jejunal GIST causing massive low gastrointestinal bleeding, requiring emergency surgery.
• This case reviews an unusual presentation of small intestinal GISTs as well as its management in an emergent context.

## 1 Introduction

Gastrointestinal stromal tumors (GISTs) are considered rare tumors. However, they are the most common mesenchymal neoplasms of the gastrointestinal tract [1,2,7]. The small intestine is the second-most frequent location where GISTs may occur, after the stomach. The jejunal GISTs are rare tumors of the digestive tract, as they account for only 0,1–3% of all gastrointestinal tumors [1,5]. The majority of GISTs are benign, but they can be malignant in 20–30% of cases.

In most cases, GISTs have an indolent clinical presentation and are discovered incidentally during endoscopic or radiologic exams or surgery. They can occasionally present as surgical emergencies such as gastrointestinal haemorrhage, intestinal obstruction or perforation [7,9].

This work presents a case of a giant GIST of the first segment of the jejunum, which presented with acute massive gastrointestinal bleeding and needed emergent surgical treatment.

The work has been reported in line with the SCARE criteria [8].

## 2 Case presentation

A 60-year-old male patient was admitted in the Emergency department of our Hospital with symptoms of abdominal pain with 2 weeks of evolution and progressive worsening and acute low gastrointestinal bleeding. The patient denied nausea, vomiting, fever and weight loss. He didn’t have personal history of medical or surgical diseases. The patient also didn’t have any history of familiar malignant disease.

**Fig. 1:**
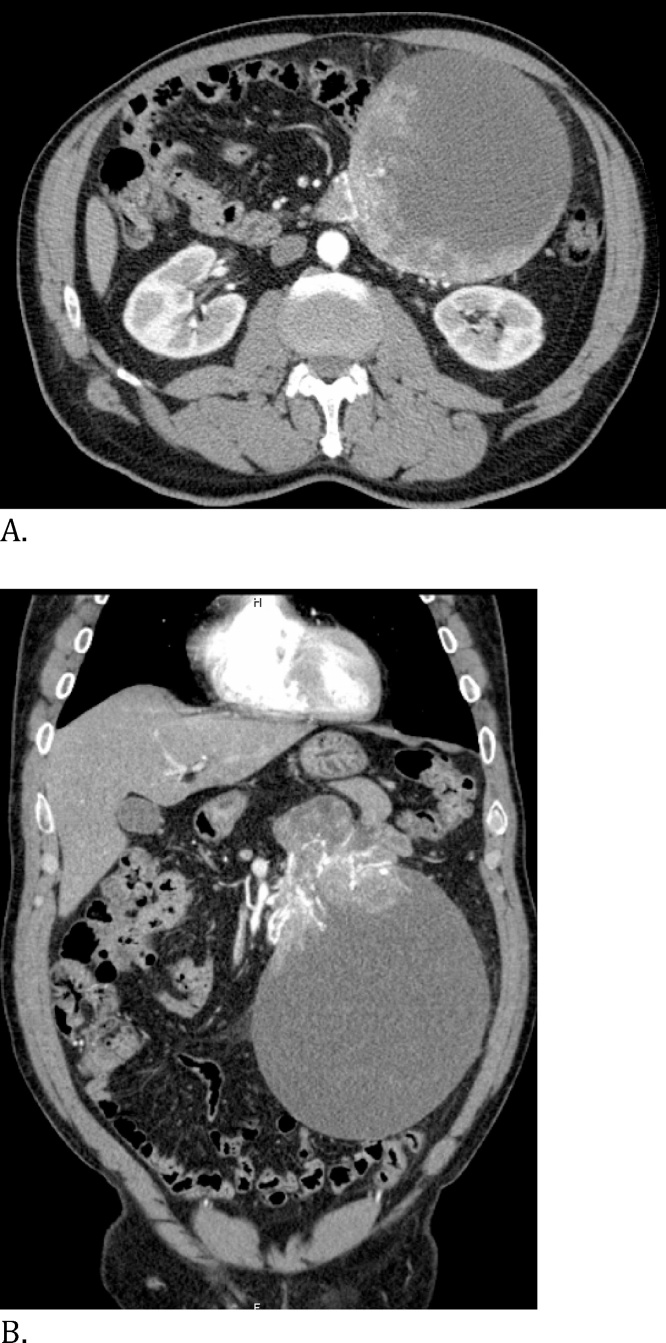
CT scan showing a large tumor arrising from the first jejunal segment: A. Sagital image; B. Coronal image.

On initial clinical observation the patient was haemodynamically stable. The examination of the abdomen revealed a palpable painless mass in the periumbilical region, left hypochondrium and left flank. There was evidence of low gastrointestinal bleeding (hematochezia). The initial haemoglobin value at admission was 133 g/dl. An emergency non-total colonoscopy showed no gastrointestinal lesions that could cause the bleeding. An Angio-CT was requested and showed a large exophytic mass arising apparently from the 4th duodenal segment and first jejunal segment with approximately 20 × 14 x 13 cm, with vascularization provided from branches from the upper mesenteric artery, with vascular dilatations inside the tumor and apparent intratumoral bleeding, without intraperitoneal bleeding (Fig. 1).

The patient’s condition deteriorated, and he became haemodynamically instable, with new episode of low gastrointestinal bleeding (hematochezia). The haemoglobin value dropped to 8,6 g/dl, requiring fluid resuscitation and blood transfusion.

**Fig. 2:**
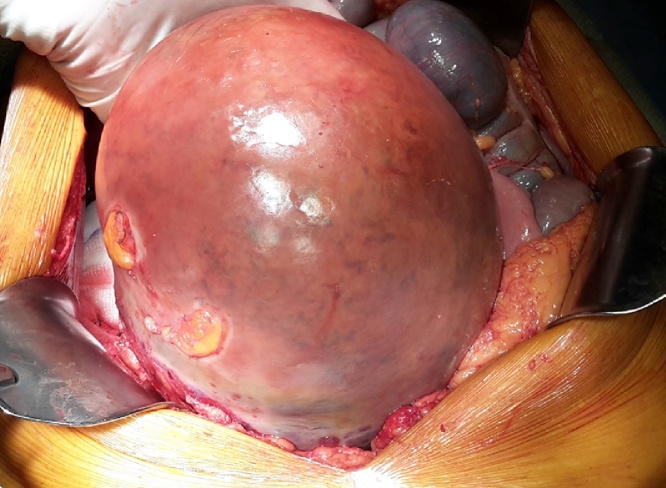
Image of the tumor found during exploratory laparotomy.

**Fig. 3:**
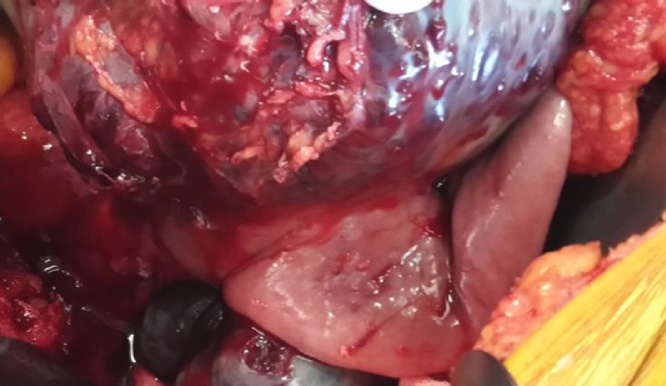
Detail of the tumoral mass arrising from the first jejunal segment.

**Fig. 4:**
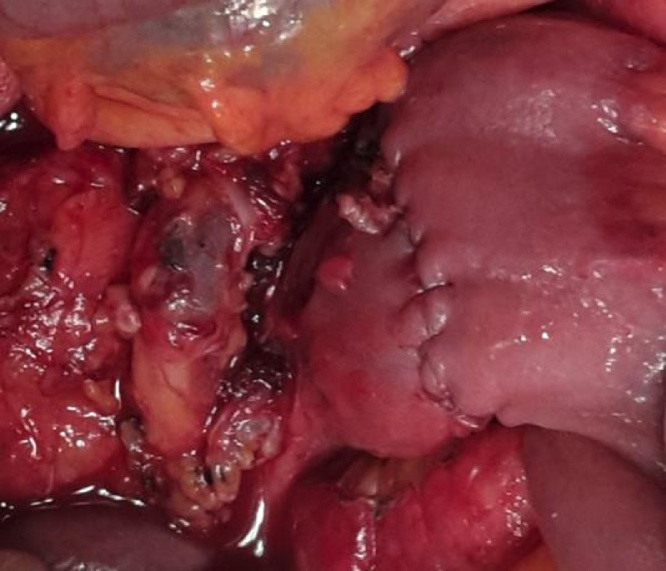
Image of the duodenal-jejunal anastomosis.

Due to haemodynamic instability the patient was submitted to emergency laparotomy, in witch a large tumor was found arising from the first jejunal segment (2 cm after duodenual-jejunal flexure) (Figs. 2e and 3 ). A segmental enterectomy was performed, resecting the mass and adjacent jejunum and the 4^th^ duodenal segment. A side-to-side manual anastomosis was performed between the 3^rd^ duodenal segment and the jejunum (Fig. 4).

The post-operative period ran without complications. The patient started oral feeding at the 7th post-operative day, after performing a gastrointestinal oral contrast study that confirmed the integrity of the anastomosis. The patient was discharged at the 11th post-operative day.

The histopathologic exam of the surgical specimen showed a gastro-intestinal stromal tumor of epithelioid cell nature, with low mitotic count (<5 per 50HPF), significant mucosal ulceration, high vascularization and tumoral necrosis. The imunohistochemical analysis revealed positivity for CD117 (c-kit) and DOG-1. The tumor was categorized as T4N0 stage IIIA. A mutation on c-KIT was found in exon 9. According to size, location and mitotic count, the tumor was categorized as high-risk GIST.

The patient was evaluated by Medical Oncology and started Imatinib therapy (400 mg per day). The follow-up continues, and the patient is free of disease recurrence for 3 years.

## 3 Discussion

Gastrointestinal stromal tumors (GISTs) are characterized by the presence of activating mutations in *KIT* or *PDGFRα* genes. Approximately 95% of GISTs are positive for CD117 [1]. The most importante prognostic factors related to these tumors are the mitotic índex, size and location [2,11].

GISTs are usually found during adulthood, and are diagnosed most frequently during the 7^th^ decade of life, with a male predominance.

These tumors can be more frequently found in the stomach (40% to 60%), followed by the small bowel (25%) [3,4,10,11]. Less frequently, they can occur in the colon, rectum, appendix, oesophagus, mesentery, omentum, or retroperitoneum. Jejunal GISTs account for 10% of GISTs arising from the gastrointestinal tract [6,12].

GISTs are usually indolent tumors. They may be discovered by symptoms, most of them nonspecific, such as abdominal pain and early satiety. They may be responsible for chronic gastrointestinal bleeding, although, in rare cases, acute massive bleeding can occur. The cause of their hemorrhagic potential is the ulceration of the mucosa [11]. The tumors of small bowel are responsible for 5–10% of occult digestive tract haemorrhage [2]. An abdominal mass may be noticeable with tumoral growth. Most patients with GISTs present variable clinical presentation, which also depends on the size and location of the tumor [1,2]. The median size of GISTs at diagnosis is 5–7 cm, although these tumors may grow over 30 cm.

The pre-operative diagnosis may be challenging and is often confirmed during surgery. In cases presented as acute gastrointestinal bleeding, endoscopic exams are usually the first modality to determine the source of bleeding. If it cannot be identified by endoscopy, a CT-scan or angiography is often performed in order to give additional information [3].

This work presents a case of a jejunal GIST that developed acute massive gastrointestinal bleeding and needed emergent surgical treatment. Small bowel GISTs are rare tumors of the gastrointestinal tract, and their presentation as acute massive haemorrhage is also rare. Once GISTs of the small intestine often show an exophytic growth pattern, they tend to be asymptomatic until they achieve large dimensions. Most cases present with chronic intraluminal gastrointestinal bleeding. Occasionally, small intestinal GISTs can present with bowel obstruction, hemoperitoneum secondary to tumor rupture, and peritonitis secondary to tumor perforation [12].

The presumptive diagnosis in this case was made before surgery, while the patient maintained haemodynamic stability, and required both endoscopic and imaging exams.

Surgical resection is the basis of treatment of GISTs and treatment with Imatinib, a tyrosine kinase inhibitor, is beneficial after resection surgery of high-risk GISTs [2,4,5,9,12]. In the case presented, the patient was submitted to radical surgical resection and then started Imatinib therapy, with a result of a follow-up period of 3 years without evidence of disease recurrence.

## 4 Conclusion

Small bowel GIST is an unusual tumour of the gastrointestinal tract and can have various clinical presentations. Its presentation as acute gastrointestinal haemorrhage is unusual, and a high index of suspicion is required for its diagnosis and management. Endoscopic and imaging exams are often indispensable to establish the pre-operative diagnosis.

This work describes a case of a giant jejunal GIST causing massive low gastrointestinal bleeding, requiring emergency surgery. The report of this case pretends to review an unusual presentation of small intestinal GISTs as well as its management in an emergent context.

## Conflicts of interest

The authors report no conflicts of interest.

## Sources of funding

There were no sponsors involved.

## Ethical approval

The study is exempt from ethnical approval in our institution.

## Consent

It was obtained a written and signed consent from the patient to publish this case.

## Author contribution

Catarina Melo – data collection, analysis and interpretation, writing the paper.

Fernando Manata – data collection, analysis and interpretation.

António Bernardes – data collection, analysis and interpretation, writing the paper.

## Registration of research studies

N/A.

## Guarantor

Catarina Melo.

António Bernardes.

## Provenance and peer review

Not commissioned externally peer reviewed.
